# Infertility is the hot topic in the 1^st^ number of International Brazilian Journal of Urology in 2024

**DOI:** 10.1590/S1677-5538.IBJU.2024.01.01

**Published:** 2024-03-18

**Affiliations:** 1 Universidade do Estado do Rio de Janeiro Unidade de Pesquisa Urogenital Rio de Janeiro RJ Brasil Unidade de Pesquisa Urogenital - Universidade do Estado do Rio de Janeiro - Uerj, Rio de Janeiro, RJ, Brasil; 2 Hospital Federal da Lagoa Rio de Janeiro RJ Brasil Serviço de Urologia, Hospital Federal da Lagoa, Rio de Janeiro, RJ, Brasil

The January-February number of Int Braz J Urol presents original contributions with a lot of interesting papers in different fields: Robotic Surgery, Prostate Cancer, Infertility, Undescended Testis, Ureteral Strictures, Penile Fracture, Percutaneous Nephrolithotomy and Prostatic Tuberculosis. The papers came from many different countries such as Brazil, Argentina, Canada, Switzerland, China, Indonesia and USA, and as usual the editor's comment highlights some of them. The editor in chief would like to highlight the following works:

Dr. Ding and collegues from China, presented in page 7 (1) a nice systematic review comparing Balloon Dilation to Non-Balloon Dilation for Access in Ultrasound-Guided Percutaneous Nephrolithotomy (PCNL) and concluded that the ultrasound-guided balloon dilatation offered several advantages in PCNL procedures. It facilitated faster access establishment, as evidenced by shorter access creation time. Additionally, it reduced the risk of kidney injury by minimizing postoperative hemoglobin loss and decreasing the need for transfusions. Moreover, it enhanced the efficiency of surgery by reducing the operation time. However, it is adequately control for confounding factors that may affect the outcomes. Therefore, further research is necessary to validate and strengthen these findings.

Dr. Hadziselimovic from Switzerland, presented in page 20 (2) a important narrative review advocating hormonal treatment to prevent adult infertility in patients diagnosed with congenital undescended testes and concluded that abnormal germ cell development in cryptorchidism is not a congenital dysgenesis but rather an endocrinopathy, preceded by hormonal imbalance and perturbation of germ cell-specific gene expression during abrogated mini- puberty. Furthermore, hormonal treatment to achieve epididymo-testicular descent as the primary choice of treatment for cryptorchidism has a long tradition in Europe. It eliminates the need for subsequent surgery, and in cases of non-responders, it facilitates orchidopexy, contributing to a reduced incidence of unilateral and the more serious bilateral complete post-surgical testicular atrophy. Therefore, the current and optimal therapeutic choice involves two steps of hormonal treatment.

Dr. Schmit and collegues from USA, presented in page 37 (3) a important study about the less qualitative multiparametric magnetic resonance imaging in prostate cancer ant the underestimation of extraprostatic extension in higher grade tumors and concluded that the negative predictive value of prostate Multiparametric magnetic resonance imaging for extraprostatic extensionmay be decreased for higher grade tumors. A detailed reference reading and image quality optimization may improve performance. However, urologists should exercise caution in nerve sparing approaches in these patients.

Dr. Wang and collegues from China, presented in page 46 (4) a nice study about the topic: Minimally invasive ureteroplasty with lingual mucosal graft for complex ureteral strictures: analysis of surgical and patient-reported outcomes and concluded that Lingual mucosal graft ureteroplasty is a safe and efficient procedure for complex ureteral reconstruction that significantly improves patient-reported HRQoL without compromising OHRQoL. Assessing patients’ quality of life enables us to monitor postoperative recovery and progress, which should be considered as one of the criteria for surgical success.

Dr. White and collegues from Prof. Ramasamy's Group in Miami - USA, presented in page 58 (5) a important study about the topic: Low-dose prednisone is an effective rescue for deteriorating semen parameters following vasovasostomy and concluded that low-dose prednisone therapy appears to be a safe and effective intervention for managing deteriorating semen parameters following vasovasostomy (VV). The observed improvements in total motile sperm count suggest the potential of prednisone to rescue patients with delayed failure after VV. Further research with larger sample sizes is warranted to confirm the safety and efficacy of low-dose prednisone as a rescue therapy in this specific patient population. Optimizing VV outcomes is crucial in male infertility, and further exploration of steroid therapy and innovative biotechnologies is warranted.

Dr. Syarif and collegues from Indonesia, presented in page 28 (6) a important systematic review about Penile fracture and concluded that tthe Most Dangerous Sexual Position that caused Penile Fracture consists in man on top position and doggy style position.

Dr. Moschovas and collegues from USA, presented in page 65 (7) a nice study about the Impacts on functional and oncological outcomes of Robotic-assisted Radical Prostatectomy 10 years after the US Preventive Service Taskforce recommendations against PSA screening and concluded that in the past years, we have witnessed a significant change in the types of patients we treat and the outcomes we are able to deliver. We are seeing younger patients with higher-grade diseases

The initial rapid rise in PSM was leveled by the move towards more partial nerve sparing. Among the increasing number of high-risk patients has led to worse functional and oncologic outcomes. some historical changes in prostate cancer diagnosis and management in the period of our study, as described in recent populational studies, the USPSTF recommendation coincided with worse outcomes of prostate cancer treatment in a population who could benefit from PSA screening at the appropriate time.

Dr. Figueiredo and collegues from Brazil, presented in page 80 (8) a nice study about the Prostate Tuberculosis and concluded that prostate tuberculosis is a disease of low suspicion and difficult diagnosis. Prostate tuberculosis manifests in well-defined six forms of clinical presentation: asymptomatic, prostate obstruction and LUTS, chronic prostatitis, recurring acute prostatitis, prostate abscess and chronic epididymitis.

The Editor-in-chief expects everyone to enjoy reading.
